# Circulating Fascin 1 as a Promising Prognostic Marker in Adrenocortical Cancer

**DOI:** 10.3389/fendo.2021.698862

**Published:** 2021-06-23

**Authors:** Giulia Cantini, Laura Fei, Letizia Canu, Giuseppina De Filpo, Tonino Ercolino, Gabriella Nesi, Massimo Mannelli, Michaela Luconi

**Affiliations:** ^1^ Endocrinology Unit, Department of Experimental and Clinical Biomedical Sciences, University of Florence, Florence, Italy; ^2^ Endocrinology Unit, Careggi University Hospital, Florence, Italy; ^3^ Department of Health Science, University of Florence, Florence, Italy

**Keywords:** fascin actin-bundling protein 1, circulating biomarker, prognosis, advanced adrenocortical carcinoma, liquid biopsy

## Abstract

Fascin-1 (FSCN1) is an actin-bundling protein associated with an invasive and aggressive phenotype of several solid carcinomas, as it is involved in cell cytoskeleton rearrangement and filopodia formation. Adrenocortical carcinoma (ACC) is a rare endocrine malignancy characterized by poor prognosis, particularly when metastatic at diagnosis. Radical resection is the only therapeutic option for ACC patients in addition to the adjuvant treatment with mitotane. Novel specific biomarkers suggestive of tumor progression to refine diagnosis and prognosis of patients with advanced ACC are urgently needed. ACC intratumoral FSCN1 has previously been suggested as a valid prognostic marker. In the present study, we identified FSCN1 in the bloodstream of a small cohort of ACC patients (n = 27), through a specific ELISA assay for human FSCN1. FSCN1 can be detected in the serum, and its circulating levels were evaluated in pre-surgery samples, which resulted to be significantly higher in ACC patients from stage I/II and stage III/IV compared with nontumoral healthy controls (HC, n = 4, FI: 5.5 ± 0.8, P<0.001, and 8.0 ± 0.5, *P* < 0.001 for stage I/II and stage III/IV group *vs* HC, respectively). In particular, FSCN1 levels were significantly higher in advanced stage versus stage I/II (22.8 ± 1.1 *vs* 15.8 ± 1.8 ng/ml, *P* < 0.005, respectively). Interestingly, circulating levels of pre-surgical FSCN1 can significantly predict tumor progression/recurrence (Log rank = 0.013), but not the overall survival (Log rank=0.317), in patients stratified in high/low PreS FSCN1. In conclusion, these findings—though very preliminary—suggest that circulating FSCN1 may represent a new minimally-invasive prognostic marker in advanced ACC, in particular when measured before surgery enables histological diagnosis.

## Introduction

Adrenocortical carcinoma (ACC) is a rare, heterogeneous endocrine tumor often characterized by poor prognosis and aggressive behavior when metastatic at diagnosis. Unfortunately, selective and effective therapies are not available, making the radical resection of the tumor mass (R0) and adjuvant administration of the adrenolytic drug mitotane (MTT) the only therapeutic strategy for ACC patients (1, 2). It has been shown that treatment with MTT improves the overall survival (OS) both in adjuvant regimen and in particular in advanced stages in association with cytotoxic agents (etoposide, doxorubicin, and cisplatinum) (1, 2). Characterization of specific and sensitive tumor markers capable of anticipating the diagnosis and also displaying a prognostic power is urgently needed, in particular for those cancers that, such as ACC, are often difficult to be studied because of their rarity.

Over the last 5 years, fascin-1 (FSCN1), a globular actin-binding protein, has emerged as an interesting novel biomarker for the most aggressive human carcinomas (3). Immunohistochemical studies have demonstrated that FSCN1 overexpression in the tumor correlates with a decreased OS and with different aspects of carcinoma invasiveness (3–7). Our previous findings have shown that FSCN1 is differentially expressed between normal adrenals and ACCs (6). More recently, we have demonstrated that the quantitative FSCN1 expression, detected by immunohistochemistry and quantitative RT-PCR in the tumor mass, may also represent a prognostic biomarker able to implement the predictive power of the current ACC histological classification (7), as high expression levels of FSCN1 positively associated with the invasive characteristics of ACC (7).

In the present study, we went further in the validation of FSCN1 biomarker in ACC, assessing whether FSCN1 was also detectable in the bloodstream of ACC patients and maintained its prognostic value, as in the tumor.

## Materials and Methods

### Patients and Ethical Approval

The study includes 27 patients affected by ACC, enrolled and evaluated at Careggi University Hospital in Florence, whose clinical characteristics are detailed in **Table 1**. All patients underwent surgical removal of the tumor mass at our hospital, and a repository of tissue specimen and blood samples has been created. Tumor samples were snap-frozen and stored at −80°C until protein extraction (7). All patients gave their written informed consent to the study, which was approved by the local ethical committee (Prot.2017-277 BIO 59/11, 27/09/2017). Matched or unmatched blood samples were selected—where available—from 9 and 26 ACC patients before (PreS) and after surgery (PostS), respectively, and stored at −20°C until the ELISA assay was performed. Two different control groups were used in the study: obese subjects with type 2 diabetes (T2D) (n = 8; mean age, 42 ± 10; mean BMI, 40.5 ± 3.1; 12.5% male) and healthy non-tumoral non-obese/T2D subjects (n = 4; mean age, 52 ± 10; mean BMI, 23.5 ± 2.3; 25% male).

**Table 1:** Clinical characteristics of the ACC cohort.

ACC PATIENT COHORT (N=27)	
**AGE (ys)**	50 ± 12 *(19-67)*
**SEX (% male)**	14 (52)
**BMI (kg/m^2^)**	25.9 ± 5.8 *(19.4-44.9)*
**SECRETION**	
**Non-secreting**	7 (26)
**Glucocorticoids**	10 (37)
**Sex steroids**	7 (26)
**Mineralcorticoids**	1 (4)
**NA**	2 (7)
**DIAMETER (cm)**	9.4 ± 5.3 *(2-18)*
**STAGE**	
**I**	6 (22)
**II**	10 (37)
**III**	8 (30)
**IV**	3 (11)
**WEISS score**	6.0 ± 1.9 *(3-8)*
**KI-67 LI (%)**	21.0 ± 18.2 *(1-70)*
**Resection status R0**	23 (85)
**R>0**	4 (15)
**Disease free survival time (months)**	43.9 ± 36.6 *(0-116)*
**Overall survival time (months)**	53.9 ± 29.9 *(9-116)*

Anthropometric data and clinical features are reported for the analyzed cohort of ACC patients. Data are expressed as mean ± SD for parametric continuous variables (age, BMI, tumor diameter, Ki67 LI %) and for Weiss, and as absolute number and percentage of patients for the other non-continuous variables (sex, stages, secretion type, resection status). Data intervals (min-max) are indicated in italics in brackets.

NA, not available.

The histopathological parameters (KI-67-LI, Weiss score) reported in **Table 1** have been evaluated by the referent pathologist (GN) at our center (8), whereas the clinical data of the ACC patients were provided by the referent endocrinologists (MM, LC, GDF).

### Tumor Lysates

Tumor samples were homogenized by mechanical disruption with Ultraturrax T10 basic IKA (Werke Gmbh & Co, Staufen, Germany) in radioimmunoprecipitation assay lysis buffer [RIPA: 20 mM Tris, pH 7.4, 150 mM NaCl, 0.5% Triton-100, 1 mM Na_3_VO_4_, 1 mM phenylmethylsulfonylfluoride (PMSF)]. After protein measurement using the Bradford method, equal amounts of proteins for each tissue sample (50 µg) were loaded onto the ELISA plate as detailed below.

### Blood Sample Collection and Separation

For each subject, blood was collected, and serum was obtained after centrifugation at 3000 rpm for 10 min at 4°C.

### ELISA Assay

FSCN1 was measured in the serum of the three groups: healthy non-tumoral non-obese/nondiabetic subjects, obese/diabetic subjects, and ACC patients using Human Fascin-1 ELISA kit (#MBS764737, MyBioSource Inc, Southern California, San Diego, USA). The sensitivity (0.469 ng/ml), the detection range (0.78-50 ng/ml), and specificity are according to the manufacturer. The intra-assay and inter-assay coefficients of variation were <8% and <10%, respectively. The obtained results were expressed as ng/ml, except for FSCN1 measured in tissue lysates, which is reported as pg/µg.

Briefly, all the buffers and reagents were equilibrated at room temperature (RT), and standard curve has been prepared and aliquoted into the plate together with the control. Blood samples previously centrifuged at 4,000 rpm at 4°C for 10 min to avoid debris, or 50 µg of tissue lysates were diluted in the specific buffer supplied in the kit and incubated in the pre-coated plate containing the anti-FSCN1 antibody for 90 min at a temperature of 37°C. After performing a series of washes with washing buffer, standards, controls, and samples were incubated with the biotinylated antibody for 60 min at 37°C. After washing, streptavidin conjugated to HRP was added for 30 min at 37°C. Following incubation in the dark with the TMB substrate for 15 to 30 min at 37°C and after adding the Stop Solution, the optical density (OD) was measured at 450 nm using a spectrophotometer (VICTOR multilabel plate reader; Perkin-Elmer). The absorbance values of the sample were interpolated on a standard curve to obtain the relative concentrations.

### Statistical Analysis

Statistical analysis was performed using SPSS software 27.0 (Statistical Package for the Social Sciences, Chicago, US) for Windows. The Kolmogorov-Smirnov’s test was used to verify normal distribution of data. Results are expressed as mean ± SE, unless otherwise stated. One-way analysis of variance (ANOVA) followed by the Dunnett’s *post hoc* test was applied for multiple comparison, whereas Student’s t-test was used for comparison of two classes of data. A P value <0.05 was considered statistically significant.

Correlation analyses were carried out Pearson’s/Spearman’s test for parametric/nonparametric continuous variables, respectively. Overall survival (OS) and disease-free survival (DFS) are defined as the probability (ranging from 0 to 1) that a patient diagnosed with the disease is still alive (OS) or is free from the disease (DFS) at a time point from surgery. Survival analysis was estimated through the Kaplan-Meier method, and differences between groups were assessed by Log rank test. Univariate and multivariate Cox regression analyses of DFS in patients stratified for the indicated factors in high and low classes for PreS FSCN1 levels and PostS FSCN1 levels were performed by SPSS.

## Results

### FSCN1 Detection in Blood Samples of ACC Patients

**Figure 1:**
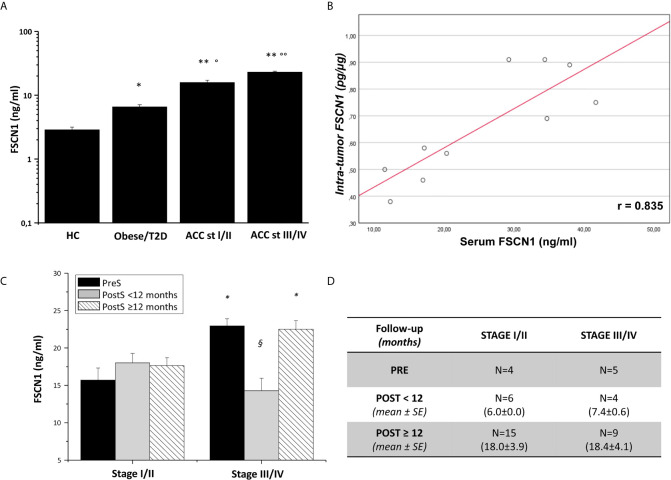
FSCN1 detection in blood samples of ACC patients. **(A)** Quantitative evaluation of FSCN1 concentrations in pre-operative serum samples of ACC patients compared to obese/diabetic and healthy controls (HC). Data are expressed as mean ± SE of circulating FSCN1 levels measured in at least n=3 independent measurements. Statistical significance obtained by One-way ANOVA followed by Dunnett’s Post-hoc test: *P < 0.01, **P < 0.0001 *vs* HC, °P<0.01, °°P<0.001 *vs* Obese/T2D. **(B)** A statistically significant positive linear correlation was found between FSCN1 detected in serum samples and in tumor tissue samples, r=0.835, R^2^ = 0.698, p=0.002, n=10. **(C)** Circulating FSCN1 levels were measured in patients with stage III/IV compared to stage I/II in pre-surgery (PreS) samples and after surgery at early (PostS <12 months) and long term (PostS ≥12 months) follow-up. Data are expressed as mean ± SE of protein levels in at least n= 3 independent measurements. Statistical significance obtained by One-way ANOVA followed by Dunnett’s Post-hoc: *P < 0.005 stage I/II *vs* III/IV; ^§^P < 0.001 PreS *vs* PostS. **(D)** The table shows the number of patients with PreS, PostS<12, and PostS≥12 samples and their follow-up time for post-surgery sampling (mean ± SE). In stage I/II group all patients had a R=0 resection status, while for stage III/IV group n=4 patients had R>0 in the PreS and PostS groups.

FSCN1 levels were measured in the serum samples of 27 ACC patients and compared with measurements obtained in the serum samples of non-tumoral obese/T2D patients (n=8) and non-tumoral/non-obese/non-T2D subjects (n=4). Clinical characteristics of ACC patients are reported in **Table 1**. Metastasis and relapse were present in 10 (37%) of 27 patients, whereas death occurred in 4 (15%) of 27 patients. Circulating FSCN1 levels in blood samples collected before surgery of ACC patients (stage I/II and stage III/IV) were significantly higher than those in the serum samples of the two cohorts of non-tumoral control subjects (fold increase, FI: 5.5 ± 0.5, P<0.0001 and 8.0 ± 0.5, P<0.0001 for stage I/II and stage III/IV group *vs* healthy controls, respectively; FI: 2.4 ± 0.4, P<0.01 and 3.5 ± 0.2, P<0.001 for stage I/II and stage III/IV group *vs* obese/T2D subjects, respectively; **Figure 1A** **)**. A statistically significant positive correlation was found between serum concentration of FSCN1 and FSCN1 levels in tumor tissues of the 10 ACC patients analyzed by ELISA technique (**Figure 1B**). When compared between stages in ACC patients, FSCN1 levels resulted significantly elevated in serum samples collected before surgery from stage III/IV patients compared with those measured for stage I/II (**Figure 1C**). Interestingly, after the surgical resection of the tumor mass, FSCN1 concentration in serum samples collected close to the surgery (PostS < 12 months from surgery) was significantly decreased in stage III/IV group only, and was raised again toward pre-surgery levels at a longer follow up (PostS ≥ 12 months from surgery). No differences in FSCN1 levels between pre- and post-surgery samples were evident in stage I/II group. The mean follow-up time interval at which serum samples were taken after surgery in ACC patients stratified in low/high stages is shown in **Figure 1D**.

### Clinical Significance of Circulating FSCN1 in ACC Patients

**Figure 2:**
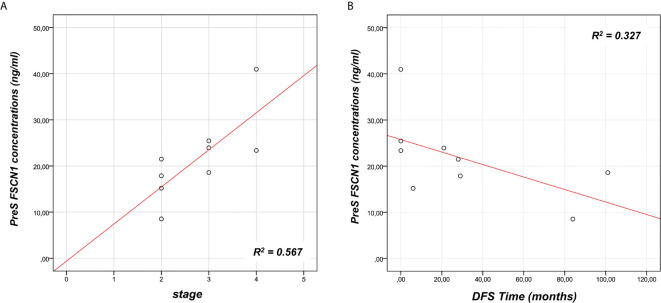
Association between FSCN1 levels and ACC parameters. A statistically significant positive linear correlation was found between pre-surgery FSCN1 concentrations (PreS) and stage - r=0.784, R^2^ = 0.567, p=0.012, n=9 **(A)**, and a statistically significant negative linear correlation with the disease-free survival time - r=- 0.695, R^2^ = 0.327, p=0.038, n=9 **(B)**.

To investigate the predictive power of circulating levels of FSCN1 in the tumor progression, we evaluated any association between pre-surgery FSCN1 concentrations and clinical characteristics of ACC patients (see **Table 1**). A statistically significant positive association was found between pre-surgery levels of FSCN1 and stage (r=0.784, p=0.012, **Figure 2A**), whereas pre-surgery FSCN1 negatively correlated with DFS time (r=-0.695, p=0.038, **Figure 2B**).

**Figure 3:**
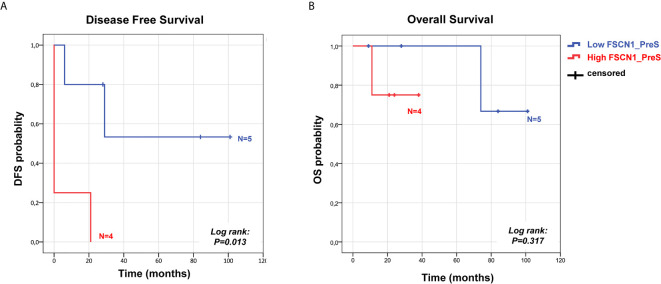
Survival predictive power of pre-surgery serum FSCN1 levels in ACC. Kaplan Meier analysis of DFS **(A)** and OS **(B)** in n=9 ACC patients stratified in two classes according to low and high FSCN1 preS serum concentrations (cut off=21.49 ng/ml, pre-surgery FSCN1 median value). Statistical significance is indicated by Log-rank.

We analyzed the prognostic power of circulating FSCN1 levels for DFS and OS by applying Kaplan-Meier analysis to ACC patients stratified by PreS FSCN1 levels. When patients were dichotomized in low (≤ 21.49 ng/ml) and high (>21.49 ng/ml) FSCN1 levels in PreS serum samples, according to the median value of pre-surgery FSCN1, FSCN1 levels significantly predicted DFS (Log-rank=0.013, **Figure 3A**), but not OS (Log-rank=0.317, **Figure 3B**). Metastasis and relapse were present in 4 (44%) of 9 patients, whereas death occurred in 2 (22%) of 9 patients. Both DFS time (49.6 ± 18.2 *vs.* 5.25 ± 5.25 months, p=0.070, Student’s t test) and OS time (59.2 ± 17.4 *vs.* 23.5 ± 5.6 months, p=0.111) were longer in the low compared to the high PreS FSCN1 level groups, although the differences were not statistically significant. PostS FSCN1 levels showed no significant predictive power for either DFS or OS when patients were stratified in high and low PostS FSCN1 levels (on the median value of PostS FSCN1 level distribution). Multivariate Cox regression analysis of DFS indicated that PreS FSCN1 remained the only statistically significant predictive factor when adjusted for PostS FSCN1 and resection status (**Table 2**).

**Table 2:** PreS levels are the best predictor of DFS.

	Univariate			Multivariate			
	HR	95%CI	P	HR		95%CI	P
**PreS FSCN1**	9.4	0.98-90.4	*0.05*	10.7		1.0-113	*0.049*
**high *vs* low**							
**PostS FSCN1**	0.3	0.04-1.9	0.181	0.2		0.3-1.8	0.161
**high *vs* low**							
**Resection status**	3.8	0.5-27.3	0.181	4.4		0.6-35.5	0.161
**R>0 *vs* R=0**							

Univariate and Multivariate Cox regression analyses of DFS in patients stratified for the indicated factors in high and low classes for PreS FSCN1 levels and PostS FSCN1 levels (according to the median value of FSCN1 distributions in serum samples drawn before and after surgery).

HR, hazard ratio; CI, confidence interval.

## Discussion

Circulating tumor markers represent a potential clue of the tumor presence obtained with minimally invasive procedures, such as blood drawn compared with intratumor markers obtained by invasive biopsies or tumor removal and, therefore, may constitute a valuable tool for patient diagnosis, prognosis, and monitoring. The most recent ACC guidelines suggest the importance of potential integration of the classical clinical parameters defined for this cancer with the molecular analysis to improve the management of this rare and aggressive endocrine tumor (9). However, despite the significant advances in characterizing the molecular intratumor landscape of ACC (10, 11), there is still a great need of developing biomarkers for early diagnosis, as the tumor histological assessment remains, until now, the gold standard for ACC diagnosis (12).

In the present paper, we demonstrated that FSCN1 levels are measurable in the bloodstream of ACC patients and are significantly higher than those measured in healthy non-tumoral subjects, reflecting the higher intratumoral levels of FSCN1 compared with normal adrenals (6, 7). Furthermore, we found a positive correlation between FSCN1 levels measured in serum and in the primary ACC mass. The levels found in non-tumoral patients in our studies are in agreement with those reported in similar studies performed in healthy subjects (13) and in other cancers (14) and lower than 10 ng/ml. Moreover, serum FSCN1 levels were significantly higher in ACC patients compared with obese/T2D patients, who have higher levels of FSCN1, probably due to kidney overload/damage associated to diabetes, as already demonstrated in renal injury associated with renal transplantation (15). These findings suggest that the high levels of FSCN1 may be indicative of an adrenal tumor, although a differential diagnostic power cannot be claimed, as circulating FSCN1 levels have not been measured in different types of adrenal tumors, such as pheochromocytoma and adrenocortical adenoma.

It is still unclear if FSCN1 release in the bloodstream is an active or a passive process from the tumor mass; and its potential role in the bloodstream is still unknown. FSCN1 was discovered as an actin-bundling protein responsible of promoting migration through its localization in cell microspikes, filopodia, and actin-based protrusions (5). Although this protein can also be expressed in normal tissues, recent studies have shown that FSCN1 is up-regulated in many types of metastatic tumors, and its expression correlates with enhanced aggressiveness, poor prognosis, and reduced survival (4, 5, 7, 16). Selective block of FSCN1 in the tumor has been recently described to inhibit the metastatic process and stimulate anti-tumor immune responses in several mouse models of solid tumors (17).

Significantly elevated concentrations of serum FSCN1 have been described in head and neck cancer (HNC) patients (18) and in non-small cell lung cancer (NSCLC) patients (14, 19) compared with healthy controls. In addition, similar to what has been found in NSCLC patients (14, 19), we showed here that serum FSCN1 levels in pre-surgery samples are significantly higher in advanced stage-patients. This is the first study also assessing serum levels of FSCN1 after the removal of the tumor mass. Although in stage I/II patients, FSCN1 levels did not significantly differ before and after surgery, in stage III/IV patients, also including R>0 masses, there was a significant drop in FSCN1 soon after the surgery followed by a return back to the levels similar to presurgery. Of note, the decrease observed did not reach FSCN1 levels as low as those in nontumoral conditions, but resembled the levels found in stage I/II. This behavior might be explained by a reduction in the active release of FSCN1 from the primary mass after its removal, which might characterize advanced ACC, followed by a return to the pre-surgery levels once the tumor progresses or relapses. The low but stable levels found in stage I/II, which, nevertheless, are higher than those found in nontumoral subjects, might be a marker of the condition of the adrenal tumor, which also remains after the mass removal. A passive leakage from the remaining epithelia or the damaged kidney may be hypothesized, as suggested by the rather high levels of circulating FSCN1 found in obese/diabetic subjects with kidney sufferance.

In our analysis, we found a positive correlation between Pre-S serum FSCN1 levels and stage, as well as a negative relationship with the DFS time, reflecting our previous findings (7) that FSCN1 expression in the tumor mass is associated with disease progression and worse prognosis. These results suggest that circulating pre-surgery FSCN1, as found in liquid biopsy, may represent a valid prognostic marker of advanced ACC.

Circulating FSCN1 may be somehow involved in tumor progression, namely in the metastatic process, as well as in tumor relapse, supported by the evidence that FSCN1 in pre-surgery samples is predictive of DFS but not of the OS. Of note, this information is obtained through a minimally invasive procedure, such as a blood sample before surgery, thus allowing the histopathological diagnosis of ACC on the removed tumor mass.

We acknowledge some limitations in our study, such as the small number of patients for PreS FSCN1 analysis, as this study was retrospectively performed, and PreS samples were not available for all the 27 patients who were tested for FSCN1 following surgery. Therefore, a different number of PreS and PostS measurements were available, and it was not always possible to evaluate FSCN1 levels in pre- and post-surgery paired serum for each patient. Other factors that might affect the analysis are the heterogeneity of ACC patients’ adjuvant treatments, which were not only limited to mitotane alone but also included EDP chemotherapy and radiotherapy (20).

Serum tumor markers have been widely used in diagnosis, prognosis, and treatment monitoring of several solid cancers (19, 21), although they might not be always sensitive enough for early diagnosis of the disease or for monitoring tumor evolution (21). A different approach reported in the literature involves the research of circulating autoantibodies against FSCN1 instead of measuring serum FSCN1 levels, even though this alternative strategy might lack sensitivity (21). However, this approach might be of a potential value in ACC, as previously demonstrated for another potential marker of ACC, FATE-1 (22).

Blood samples are a rich source of tumor material and the liquid biopsy is rapidly emerging as a minimal-invasive and powerful tool for diagnosis and prognosis of solid cancers.

In conclusion, our preliminary findings suggest that the measurements of circulating FSCN1 may represent an innovative minimally invasive marker of advanced ACC, in particular when measured before surgery can provide material for the histological diagnosis. The proposed marker can provide additional information to be used to refine ACC patients’ management. Validation of the data of our preliminary study in extended cohorts of ACC patients is mandatory.

## Data Availability Statement

The raw data supporting the conclusions of this article will be made available by the authors, without undue reservation.

## Ethics Statement

The studies involving human participants were reviewed and approved by the local ethical committee (Prot.2017-277 BIO 59/11, 27/09/2017). The patients/participants provided their written informed consent to participate in this study.

## Author Contributions

GC: study concept and design, analysis and interpretation, drafting of the manuscript, writing—review and editing, and final approval of the version to be published. LF and TE: substantial contributions to data acquisition. LC and GDF: data curation. GN: data interpretation. MM: substantial contributions to critical revision of the manuscript, and final approval of the version to be published. ML: study concept and design, supervision, writing—review and editing, final approval of the version to be published, agreement with all aspects of the work, and funding acquisition. All authors: final approval of the version to be published, and agreement with all aspects of the work. All authors contributed to the article and approved the submitted version.

## Funding

This work was supported by Associazione Italiana Ricerca sul Cancro (AIRC) Investigator Grant to ML (grant IG2015-17691); Seventh Framework Program (FP7/2007-2013) under grant agreement 259735 ENS@T-Cancer.

## Conflict of Interest

The authors declare that the research was conducted in the absence of any commercial or financial relationships that could be construed as a potential conflict of interest.
